# Moderators of School-Based Physical Activity Interventions on Cardiorespiratory Endurance in Primary School-Aged Children: A Meta-Regression

**DOI:** 10.3390/ijerph15081764

**Published:** 2018-08-16

**Authors:** Ryan D. Burns, Timothy A. Brusseau, You Fu

**Affiliations:** 1 Department of Health, Kinesiology, and Recreation, University of Utah, Salt Lake City, UT 84112, USA; tim.brusseau@utah.edu; 2 School of Community Health Sciences, University of Nevada Reno, Reno, NV 89557, USA; youf@unr.edu

**Keywords:** aerobic fitness, exercise, health, meta-analysis, youth

## Abstract

The purpose of this study was to examine potential moderators of school-based physical activity interventions on cariorespiratory endurance in primary school-aged children using meta-regression. An Internet search with several databases was employed, extracting school-based pediatric physical activity intervention studies published within the past 30 years. Studies were included if there was a control or comparison group, if the study sample included primary school-aged children, if the targeted outcome of cardiorespiratory endurance was objectively assessed, if the intervention was at least partially school-based, and if the effect estimate’s variability was reported. An inverse-variance random effects meta-regression was employed using the primary predictors of component number (single component or multi-component) and intervention length using 20 extracted studies with 23 total effects. The overall pooled effect on cardiorespiratory endurance was statistically significant (Hedges’ g = 0.30, 95% C.I.: 0.19–0.40; *p* < 0.001). Using random effects meta-regression, neither component number (b = −0.09, 95% C.I.: −0.40–0.23; *p* = 0.560) or intervention length (b = 0.001, 95% C.I.: −0.002–0.004; *p* = 0.427) yielded a significant modifying effect on cardiorespiratory endurance. School-based physical activity interventions have a significant pooled effect on cardiorespiratory endurance in primary school-aged children. Component number and intervention length does not modify this effect, suggesting other sources for between-study heterogeneity.

## 1. Introduction

Physical activity is a modifiable health behavior that has numerous benefits for children, including decreased risk for non-communicable cardio-metabolic disease [1], improved cognitive functioning [2], and improved emotional well-being [3]. Despite these benefits, it has repeatedly been shown that children do not meet the recommended guideline of 60 min of physical activity per day [4,5]. To address this important public health issue, over the past couple of decades, school-based physical activity interventions have been a popular strategy to facilitate children meeting the 60-min per day guideline and to develop the knowledge and skills to be physically active throughout their lifespan [6,7].

School-based physical activity interventions primarily focus on providing additional, enhanced, or expanded physical activity opportunities during specific school components, such as during physical education or by incorporating activity breaks in the academic classroom [8,9,10]. Increasingly, interventions have been incorporating a multi-component approach, such as Comprehensive School Physical Activity Programming, which use all available resources throughout the school day to improve physical activity behaviors and decrease sedentary times in children and adolescents [11,12]. Various components targeted by these interventions include physical education, during the academic classroom, recess, before/after school, and the family and community. Many single and multicomponent interventions have been successful in being implemented in schools enrolling different populations of youth (e.g., low-income, ethnic/racial minorities) with respective interventions aligning with various established theoretical frameworks (e.g., social cognitive theory, ecological model) [13,14,15]. However, it is unclear if multicomponent intervention approaches significantly yield greater efficacy or effectiveness compared to single component intervention approaches, and if other characteristics of said interventions, such as intervention length, modify program efficacy. Therefore, given the relatively large pool of published work in this area, much can be learned by testing these physical activity intervention characteristics as potential moderators using meta-regression statistical methods.

Most research examining the efficacy of school-based physical activity interventions use the behavior of physical activity as a primary outcome of interest [16,17]. Inherently, this is to be expected as it is this health behavior that is targeted, however its assessment not without limitation, given the varying evidence for construct validity for some of the employed instruments (e.g., questionnaires, systematic observation) and the numerous potential confounders that may influence observed results using more objective instruments such as accelerometers (e.g., accelerometer epoch length, accelerometer wear time) [18,19,20]. Additionally, physical activity is usually only assessed across one week or school week, which can be further confounded by weather, school scheduling, etc. [21,22,23]. Indeed, systematic reviews and meta-analyses exploring the pooled effect of physical activity interventions have often yielded null findings or effects that are small in magnitude [24,25], which may be partially due to the aforementioned confounding factors in the assessment of physical activity behaviors. Therefore, other constructs can be used to establish the efficacy or effectiveness of school-based physical activity interventions. Additional important outcomes of physical activity interventions are the domains of health-related fitness: body composition, cardiorespiratory endurance, muscular strength and endurance, and flexibility.

Health-related fitness consists of five domains consisting of body composition, cardiorespiratory endurance, muscular strength and endurance, and flexibility. Cardiorespiratory endurance is a domain that has established moderate links with health outcomes in the pediatric population [26,27]. Cardiorespiratory endurance is the ability of the circulatory and respiratory systems to supply oxygen during prolonged physical activity and is a domain often assessed in national fitness test batteries (e.g., Fitnessgram) using measured or estimated relative aerobic power (VO_2 Peak_) as the metric of assessment [28]. Research has shown independent relationships between cardiorespiratory endurance and cardio-metabolic risk in children and adolescents, independent of body composition levels [29,30]. Indeed, because body composition (e.g., percent body fat, body mass index (BMI)) is often cofounded by other energy balance behaviors such as diet and out-of-school physical activity [31,32], cardiorespiratory endurance may be a more practical domain of health-related fitness to assess within the context of school-based intervention assessment and may reflect habitual physical activity sustained over periods of time [33]. Additionally, because cardiorespiratory endurance is a physiological trait, it can be easily targeted in its assessment as evidenced by the strong construct validity of various established lab and field tests utilized in pediatric fitness surveillance research [34,35]. Moderating influences of school-based physical activity interventions have been explored in adolescents, however, to the authors’ knowledge, no study has quantified various moderating influences of school-based physical activity interventions on the health-related fitness domain of cardiorespiratory endurance in children. Therefore, the purpose of this study was to examine the modifying influence of intervention component number and intervention length on the effect of school-based physical activity interventions on cardiorespiratory endurance in children using random effects meta-regression.

## 2. Materials and Methods

### 2.1. Literature Search Strategy

An internet search with several databases was performed (MEDLINE (PubMed), SCOPUS, EMBASE, SportDiscus) using the keywords “Aerobic Fitness”, “Cardiorespiratory Endurance”, “Children”, “Exercise”, “Interventions”, “Physical Activity”, and “School”. The Boolean operator ‘AND’ was used for combinations among keywords. The initial selection was based on the titles of manuscripts and the abstracts. Two authors performed separate independent searches and assessed extracted studies for relevance and eligibility. Discrepancies in the initial extracted studies were settled by the third author. Discrepancies were settled by consensus and the initial set of extracted studies was assessed further for eligibility.

### 2.2. Eligibility Criteria

**Figure 1:**
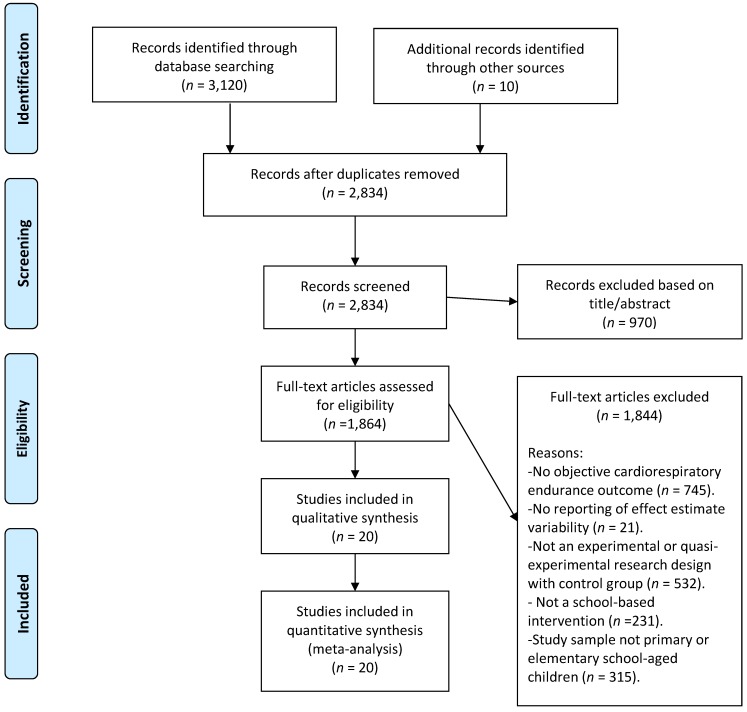
Flowchart of study extraction and inclusion.

A flow chart communicating the study search and extraction procedures is provided in Figure 1. After extraction of the original identified studies, studies were included in the meta-analysis based on having an assessment of cardiorespiratory endurance as an outcome, employing a controlled experimental or quasi-experimental research design (intervention efficacy or effectiveness study) within a school setting, and the reporting of the effect estimate’s variability in the form of standard errors or standard deviations. Studies were included if they had a direct measure of aerobic power, an aerobic power estimate, or a proxy performance assessment (e.g., shuttle run laps) as a primary or secondary outcome. Studies were not included if the primary intervention was in an “after-school” or “community” setting. Studies employing a pre-test/post-test design with no control or comparison group were also eliminated. Finally, interventions targeting just girls or interventions targeting specific healthy children populations (e.g., low-income) were included in the analysis, however interventions targeting youth with physical disabilities were eliminated. Interventions targeting adolescents in middle or secondary schools were excluded. There was consensus between two primary reviewers on the final twenty extracted studies used for the meta-analysis.

### 2.3. Type of Participants

Participants were children enrolled in primary/elementary schools participating in a school-based physical activity intervention. Interventions targeting a specific sex or children who were overweight or obese were included. Given the information presented in the included studies, all subjects were provided informed consent and signed assent forms, with parents or guardians signing consent forms prior to data collection.

### 2.4. Types of Physical Activity Interventions

To be included in the meta-analysis, at least some aspect of the intervention must have been implemented during school hours. Both single and multicomponent physical activity interventions were included. Interventions were included if they incorporated high-intensity interval training (HIIT) but were excluded if resistance training was the only employed exercise modality, given these interventions target the muscular strength and endurance domains of health-related fitness. Intervention length was not a parameter used for study inclusion or exclusion.

### 2.5. Outcome Measures

Studies were included having a direct measure of aerobic power (measured VO_2 Peak_), an estimate of aerobic power calculated from a validated prediction algorithm, or a performance proxy assessment, such as shuttle run laps or one-mile run/walk times. Self-reported measures or tests assessing anaerobic performance were excluded. Cardiorespiratory endurance did not have to be a primary outcome of interest to be included in the meta-analysis.

### 2.6. Calculation of Standardized Mean Differences

For each selected study, three sources of information were used to calculate the effect sizes or standardized mean differences (SMDs). These sources of information included: (1) the intervention and control group’s sample sizes, (2) the intervention and control group’s mean differences between pre-test and post-test on the respective cardiorespiratory endurance assessment, and (3) the standard deviation of the intervention and control group’s mean differences on the respective cardiorespiratory endurance assessment.

### 2.7. Data Processing

Primary predictors in the employed meta-regression were component number and intervention length. The outcome variable was the standardized mean differences at the study level. The predictor of component number (single, multiple) was a dichotomized categorical variable. The referent was single component interventions. Intervention length was examined on the continuous measurement scale and was scaled in weeks. If the length of the intervention in a manuscript was communicated in months, the number was multiplied by 4.35 to obtain the approximate number of weeks. Sex was not included as a predictor because of the small number of single sex-controlled school-based interventions targeting cardiorespiratory endurance.

### 2.8. Statistical Analysis

For three of the 20 extracted studies, the reporting of the results was either stratified by sex (boy, girl) or by body composition status (normal weight, overweight/obese). These studies were treated as separate sub-studies clustered within a larger study. This methodology yielded 23 separate standardized mean differences. Because the number of higher-level clusters was small, we decided not to statistically adjust for this clustering of results. The employed meta-analysis tested the null hypothesis that the overall effect (Hedges’ g) of school-based physical activity interventions on improving cardiorespiratory endurance was zero. The Der Simonian and Laird random effects model was employed. Random effects models take into account both between-study variance and within-study sampling error. Studies were weighted based on inverse variance, which included both within-study sampling error and between-study variance (i.e., heterogeneity). Individual study effects and the overall summary effect were reported within a Forest Plot with corresponding 95% Confidence Intervals.

Heterogeneity across the studies was quantitatively assessed using Cochran’s Q test (with alpha set at *p* ≤ 0.10) and the I^2^ statistic [36]. The magnitude of between-study heterogeneity was determined to be small if I^2^ < 50%, moderate if I^2^ = 50%–75%, and large if I^2^ > 75% [36]. Publication bias was assessed via visual inspection of Funnel Plots and was statistically assessed using the Egger linear regression test and a Galbraith plot [37]. Publication bias usually occurs when studies with relatively smaller sample sizes are published due to the presence of significant findings, which in turn may cause a mis-representativeness of available evidence and may distort the observed overall pooled effect. Funnel Plots were determined to be asymmetrical if the intercepts from the Egger regression model and Galbraith plot significantly deviated from zero [37]. The Funnel Plot was obtained using STATA’s “metafunnel” command and the Egger regression parameter estimates with Galbraith plots were obtained using STATA’s “metabias” command.

To explore calculated between-study heterogeneity, a random effects meta-regression was performed using STATA’s “metareg” command. Standardized mean differences were regressed onto intervention component number and intervention length using residual maximum likelihood (REML) to estimate the between-study component of variance. Calculated parameter estimates (b-coefficients) with corresponding 95% Confidence Intervals were reported. A scatterplot and line of best fit was presented displaying the linear relationship between the standardized mean differences and the continuous length predictor.

A post hoc sensitivity analysis was conducted, removing a single study from the meta-analysis per iteration to determine if the parameter estimates would significantly change compared to if that respective study was included. Any adjusted pooled effects and coefficients were reported and discussed. This procedure was conducted to ensure that no single one study significantly influenced the results.

If the aforementioned meta-analysis was found to contain publication bias, the nonparametric trim and fill method was employed using STATA’s “metatrim” command. This method estimates the number of missing studies needed to correct for publication bias and provides an updated Funnel Plot and pooled fixed and random effect estimates. It is cautioned that this procedure only fills hypothetical studies in order to yield a more symmetrical Funnel Plot. Results of this analysis are reported in the supplementary material. Alpha level was set at *p* < 0.05 and all analyses were conducted using the STATA v.15.0 statistical software package (College Station, TX, USA).

## 3. Results

### 3.1. Sample Characteristics

A total of 20 studies were extracted for the meta-analysis. Sample and intervention characteristics are reported in Table 1. Across the 20 studies, 10,779 (4879 control, 5900 intervention) primary school-aged children were recruited and analyzed on cardiorespiratory endurance outcomes. Studies were conducted in Australia (*n* = 3), Canada (*n* = 1), China (*n* = 1), France (*n* = 1), Iceland (*n* = 1), Ireland (*n* = 1), The Netherlands (*n* = 1), Norway (*n* = 1), United Kingdom (*n* = 2), United States (*n* = 6), Sweden (*n* = 1), and Switzerland (*n* = 1). Three studies included children from low-income schools. All children were recruited from primary or elementary grade levels/schools [38,39,40,41,42,43,44,45,46,47,48,49,50,51,52,53,54,55,56,57].

### 3.2. Intervention Characteristics

Of the 20 extracted studies, 13 were randomized control trials (RCTs) [38,40,41,43,44,47,48,49,50,52,54,56,57], and 10 were multicomponent [38,39,41,44,48,49,50,52,54,56]. Intervention lengths ranged from six weeks to three years in duration. Eight of the 20 interventions included expanding, extending, or enhancing the quality of physical education lessons [39,41,43,49,50,52,54,55]. Six of the 20 interventions included academic classroom activity breaks [40,42,46,50,52,56], and three included enhanced health education lessons [44,45,48]. Five of the interventions expanded, extended, or enhanced physical activity opportunities during the school day that were not specific to physical education or during academic lessons [38,47,48,51,57].

### 3.3. Assessments

All extracted studies assessed cardiorespiratory endurance [38,39,40,41,42,43,44,45,46,47,48,49,50,51,52,53,54,55,56,57]. Two studies directly measured aerobic power using maximal progressive tests on the cycle ergometer or treadmill [48,53]. Most extracted studies used the 20-m multistage fitness test to assess cardiorespiratory endurance (*n* = 9) [38,40,41,45,50,51,52,56,57]. Three studies used the Progressive Aerobic Cardiovascular Endurance Run (PACER) test [43,46,47], and four studies used timed runs for assessment [39,49,54,55]. One study used the Eurofit submaximal cycle ergometry test [44], and one study used the 10-m Andersen test for assessment [42].

### 3.4. Meta-Analysis

**Figure 2:**
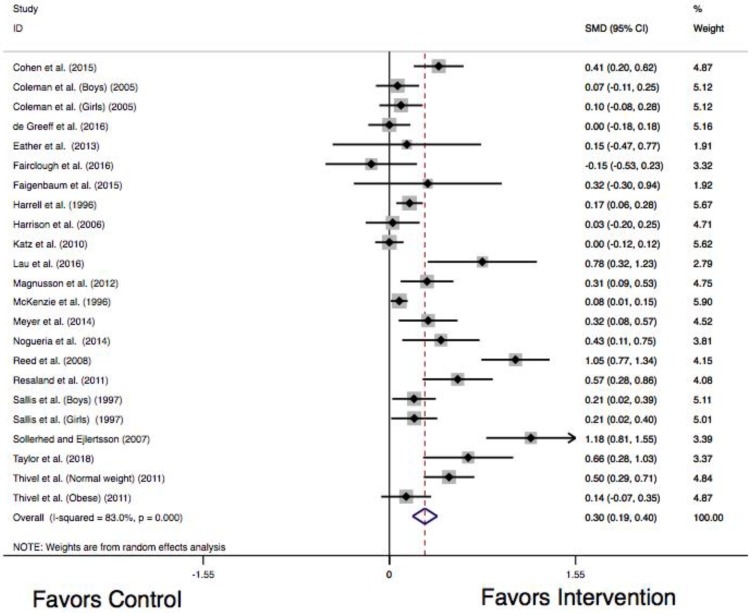
Forest plot communicating individual and pooled standardized mean differences for cardiorespiratory endurance using the random effects Der Simonian and Laird model. Note: SMD stands for weighted standardized mean differences; 95% C.I. stands for 95% Confidence Interval.

**Figure 3:**
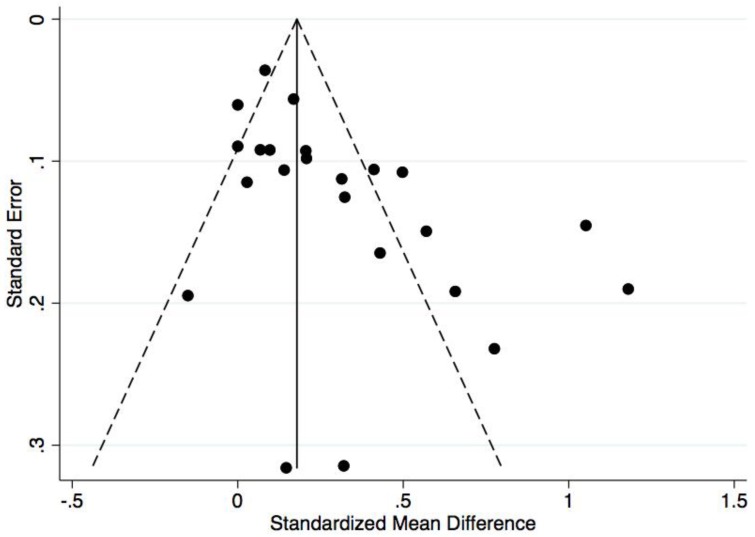
Funnel plot showing the standard error of mean differences on the y-axis and the standardized mean differences on the x-axis for cardiorespiratory endurance.

**Figure 4:**
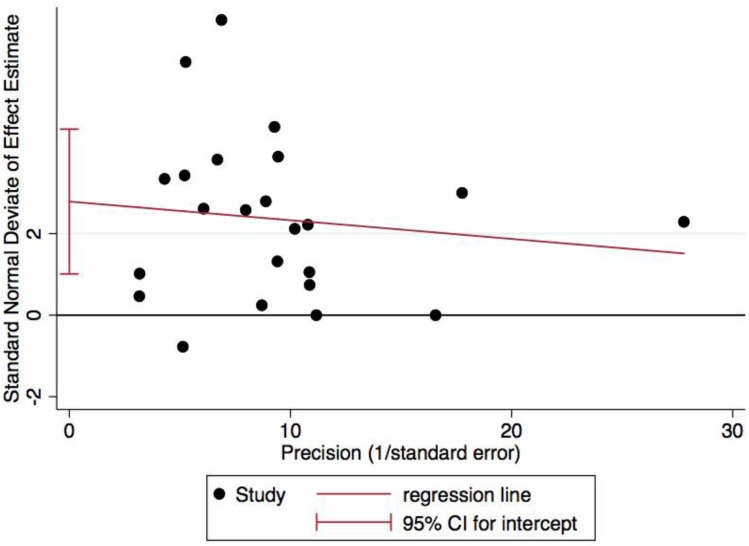
Galbraith plot showing the standard normal deviate on the y-axis and study precision (1/standard error) on the x-axis for cardiorespiratory endurance. Note: SND stands for standard normal deviate; statistically significant intercept indicates presence of small study effects.

The results of the random effects meta-analysis are presented in Figure 2. The overall pooled effect on cardiorespiratory endurance was statistically significant (Hedges’ g = 0.30, 95% C.I.: 0.19–0.40; *p* < 0.001), indicating an overall small pooled effect size. Heterogeneity across the studies was considered high (I^2^ = 83%, χ^2^(22) = 129.5, *p* < 0.001). Visual inspection of the Funnel Plot (Figure 3) suggests asymmetry. This was supported using Egger regression, as the constant was statistically different from zero (b_0_ = 2.78, 95% C.I.: 1.01–4.56; *p* = 0.004), suggesting the presence of publication bias. Figure 4 presents a Galbraith plot aligning with the Egger regression model, displaying the standard normal deviate on the y-axis and study precision on the x-axis. The 95% Confidence Interval does not cross the x-axis, supporting the presence of small study effects.

### 3.5. Meta-Regression

**Figure 5:**
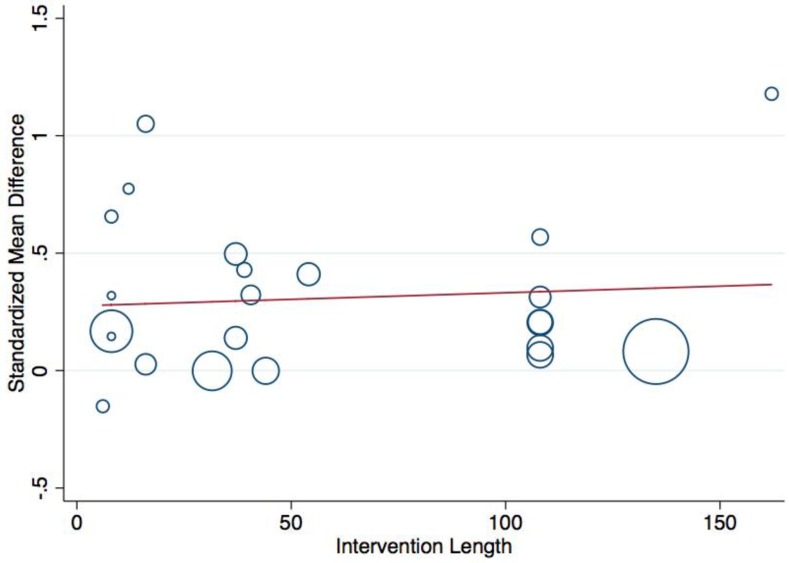
Scatterplot and line of best fit showing the study-level linear relationship between standardized mean difference and intervention length (in weeks). Note: Size of circle indicates magnitude of study precision (1/standard error).

The results of the meta-regression analysis are presented in Table 2. Neither component number or intervention length were significant predictors of the obtained aggregate-level standardized mean differences. Figure 5 visually displays the linear relationship between the standardized mean differences and the continuous intervention length predictor variable. The size of the circles corresponds to the magnitude of study precision (i.e., 1/standard error).

### 3.6. Sensitivity Analysis

A sensitivity analysis was performed removing one study from the analysis per iteration to observe any significant changes in the parameter estimates. Removal of no one study significantly changed Hedges g, the Egger bias coefficient, or altered the results from the meta-regression. These results support that the obtained results from the meta-analysis and meta-regression are relatively robust.

### 3.7. Trim and Fill

Using the nonparametric trim and fill method, seven studies would theoretically need to be replaced to correct for publication bias. This would lower the overall pooled effect to a smaller estimate using the random effects model (Hedges’ g = 0.132, 95% C.I.: 0.016–0.249; *p* = 0.026). A table with the filled studies with their respective theoretical effect estimates, in addition to a revised Funnel Plot is provided in the Supplementary Material.

## 4. Discussion

The purpose of this study was to examine the moderators of school-based physical activity interventions on the health-related fitness domain of cardiorespiratory endurance in primary school-aged children. The results suggest a significant pooled effect of school-based interventions on cardiorespiratory endurance, however neither intervention component number nor intervention length modified this effect. The results from this review could manifest important information for researchers and practitioners devising effective interventions in primary school-aged children. A discussion on the potential mechanisms for the observed relationships, in addition to practical implications are provided.

A salient finding from the current analysis was that school-based physical activity interventions have a pooled effect on cardiorespiratory endurance levels in primary school-aged children. This effect was characterized as small in magnitude. Past systematic reviews have communicated that interventions tend to be more effective in improving physical activity in adolescents, especially multicomponent interventions [58]. In children, there has been less evidence for efficacy, especially interventions targeting low-income communities [58]. Specifically, in school settings, a meta-analysis conducted by Russ et al. [25] found statistically significant but small pooled effects for multicomponent interventions to increase physical activity in children and adolescents. Reasons for insignificant or small efficacy evidence are unclear within the pediatric population. Especially in young children, there are many confounding variables that could affect the targeted outcomes of physical activity interventions over time. Motivational constructs such as physical activity enjoyment may play an important role in sustaining elevated physical activity behaviors in children [59]. It could be argued that long duration interventions tend to lose novelty and thus lower interest and enjoyment in younger children [59]. Additionally, constructs such as goal-setting, which is often employed in various forms in school-based and community settings to promote physical activity [60,61], may be too difficult and too abstract for younger children to grasp, especially in its relationship to improve health and wellbeing [62]. Therefore, interventions that set individual-level or group-level goals for physical activity or for specific domains of health-related fitness may not be as effective in younger children compared to older adolescents [63]. However, at least in the current review, controlled interventions yielded a clear significant but small effect on cardiorespiratory endurance levels. Despite the small effect, it was robust given the results from the sensitivity analysis and inclusion of theoretical studies to correct for publication bias.

Establishing efficacy of school-based interventions to improve cardiorespiratory endurance is an important finding that supports the implementation of these interventions in children. Improvements in cardiorespiratory endurance may be clinically and practically important, given that cardiorespiratory endurance is a trait with established links to cardio-metabolic health and cognitive functioning [64,65,66]. Despite these positive findings, like many past meta-analyses examining physical activity interventions [67], there was high observed heterogeneity across the included studies. This phenomenon, often observed in pediatric behavioral interventions, was addressed by performing meta-regression on the potential moderators of intervention component number and intervention length.

Using meta-regression, it was found that there was no modifying effect of intervention component number on cardiorespiratory endurance levels. As stated previously, it has been recommended that school-based interventions adopt a multicomponent approach to increase physical activity during the school day [11]. Systematic reviews and meta-analysis support this recommendation in older adolescents, as interventions using the multicomponent paradigm have yielded greater effects compared to single components programs for increasing physical activity levels [58]. Specific components of programs such as Comprehensive School Physical Activity Programming (CSPAP) include promotion of physical activity during school, before and after school, staff wellness and family and community engagement [68,69]. Multicomponent programs have been recommended across the globe in countries such as Ireland, Finland, France, Germany, the United States, and Switzerland [68,69]. Although these types of physical activity programs have been recommended, adoption and evaluation have been sparse because of limited resources including cost, time, and availability of quality trained staff to implement the program [25]. Interestingly, the results of the current analysis suggest that multicomponent interventions did not have a benefit over single component interventions for improving primary school-aged children’s cardiorespiratory endurance levels.

Two of the studies reporting the greatest effect in this review implemented two different types of physical activity programming. Reed et al. [52] implemented a multicomponent intervention called “Action Schools! BC” where six “Action Zones” were targeted across a one-year intervention. These “Action Zones” are similar to those incorporated into CSPAP, but also included additional components such as “School Spirit”. Cardiorespiratory endurance was the outcome with the greatest change at the end of the intervention, with the authors reporting 20.4% unadjusted difference (increase) in change in shuttle run laps [58]. Conversely, Sollerhed and Ejlertsson [55] reported similar strong effects on cardiorespiratory endurance, however this particular intervention study used a single component approach, merely expanding the frequency of physical education lessons from two to four lessons per week across a three-year intervention. Given the drastically different methodological paradigms from the two aforementioned interventions, it can be suggested that in at least primary school-aged children, single component interventions, such as merely increasing the time or frequency of physical education, can be just as effective as implementing large-scale multi-component programs if executed efficiently and effectively. What may be of greater influence is the quality of the staff training and the degree of intervention program fidelity [70]. Additionally, specific programming characteristics such as the specific intensity of physical activity and frequency may also be potential moderators [71]. These potential effects should be explored with additional research.

Another important finding from the analysis was that there was no linear relationship between intervention length and the observed standardized mean differences. In a systematic review with meta-analysis conducted by Minatto et al. [72], examining the effect of school-based interventions on cardiorespiratory endurance in older adolescents, it was found that interventions ranging from 13–24 weeks in duration yielded greater improvements in cardiorespiratory endurance compared to interventions of shorter and longer durations. There could be an optimal intervention length to elicit improvements in cardiorespiratory endurance. The findings from Minatto et al. [72] support this, however the mechanisms for variation in effect due to length are unknown. As stated previously, longer length interventions may attenuate novelty and interest in younger children, which may affect motivational constructs such as physical activity enjoyment [59]. Because habitual physical activity is needed to improve cardiorespiratory endurance, decreases in the latter construct may be found with interventions of longer length. Conversely, very short duration interventions may not be long enough to elicit the physiological responses needed to improve aerobic capacity in younger children. Despite these theoretical mechanisms, intervention length did not relate to the observed effects at the study level. This could suggest other moderators may influence cardiorespiratory endurance, such as physical activity intensity and the duration of specific physical activity sessions within each respective intervention. Interestingly, in adolescents, Minatto et al. [72] did observe trends among the intensity of physical activity, weekly frequency of physical activity sessions and the observed standardized effects, however, these moderators were not tested statistically and there are many studies that do not report these specific characteristics when communicating the intervention; therefore, it is difficult to conclude whether these would be significant moderators. Finally, Crimarco et al. [73] found a relationship between school attendance and amotivation for physical activity. It could be that children who are not motivated to participate in physical activity have lower school attendance and thus fail to participate in the opportunities provided by a respective program. Especially in children who already have higher levels of amotivation, school-based programming may lead to a decrease in attendance and attenuate overall program efficacy. Indeed, the tailoring of interventions to elicit the most favorable outcomes is inherently complex; however, given the results of this study, it seems that in younger primary school-aged children, intervention length does not significantly modify the efficacy of school-based physical activity interventions on cardiorespiratory endurance.

These findings are important for both researchers and practitioners who are planning and implementing physical activity programming in schools with a goal of improving cardiorespiratory endurance. With the limited physical activity opportunities in schools and the challenge/cost of long interventions, this meta-regression highlights the impact even short single component interventions might have. More specifically, the enhancement of physical education or the expansion of classroom physical activity may have the ability to positively impact cardiorespiratory endurance in primary school-aged children. The addition of classroom-based activity or the improvements that might be made in physical education classes have little cost associated and the time is also minimal. Furthermore, practitioners may be able to have a positive change in a short period of time (e.g., six weeks), thus making programming appear more doable for teachers. If physical education teachers know that they can make meaningful change over the course of a unit (instead of the entire year), they may be more likely to implement programming. Similarly, if a research paraprofessional or a classroom teacher realizes that a shorter intervention can have a meaningful impact, they may be more likely to try something new and once they try it and have success, perhaps they are more likely to continue with the program in the long term.

There are limitations to this study that should be considered before the results can be generalized. First, the inclusion criteria for the meta-analysis consisted of only school-aged children enrolled in primary or elementary school; therefore, the results do not generalize to older adolescents or younger preschool-aged children. Second, other moderators were not included in the meta-regression, such as sex, age, initial levels of health-related fitness, and type of assessment employed for cardiorespiratory endurance. These factors could have been significant moderators, however there was not enough information available to include them in the meta-regression model. Third, process information concerning intervention fidelity was not collected or analyzed in this review; variation in intervention implementation fidelity may have confounded the results. Finally, only cardiorespiratory endurance was targeted as the outcome of this review; overall intervention efficacy should not be solely based on variation of this construct but should only be considered in context with other observed outcomes in a respective study.

## 5. Conclusions

In conclusion, school-based physical activity interventions have a significant effect on cardiorespiratory endurance in primary school-aged children. Using meta-regression, it was found that neither intervention component number nor intervention length modified this effect; therefore, other factors may be responsible for between-study heterogeneity. Practically, the results of this analysis suggest that single component interventions could be just as effective as multicomponent interventions for improving cardiorespiratory endurance. Additionally, because intervention length was not a significant effect modifier, the paradigm that long duration programs may yield greater health benefits should be in question. In the context of improving cardiorespiratory endurance, factors other than component number and intervention length should be targeted to yield acceptable physical activity program efficacy.

## Figures and Tables

**Table 1:** Methodological summary of the 20 selected studies.

Study	Study Location	Sample Size	Sample Characteristics	Intervention Length	Intervention Characteristics	Cardiorespiratory Endurance Assessment
Cohen et al. (2015) [38]	Australia	*n* = 208 (control) *n* = 162 (intervention)	Low-income children	12 months	RCT; multicomponent; physical activity and fundamental motor skill focus	20-m multistage fitness test
Coleman et al. (2005) ^†^ [39]	United States	*n* = 473 (control) *n* = 423 (intervention)	Low-income children	24 months	Non-RCT; multicomponent; enhanced physical education and cafeteria nutrition, parent education	Nine-minute timed run
De Greeff et al. (2016) [40]	The Netherlands	*n* = 250 (control) *n* = 249 (intervention)	Primary school-aged children	24 months	RCT; single component; physically active academic lessons	20-m multistage fitness test
Eather et al. (2013) [41]	Australia	*n* = 16 (control) *n* = 27 (intervention)	Primary school-aged children	Eight weeks	RCT; multicomponent; physical education, recess, home fitness program	20-m multistage fitness test
Fairclough et al. (2016) [42]	United Kingdom	*n* = 48 (control) *n* = 59 (intervention)	Primary school-aged children	Six weeks	Non-RCT; single component; structured class-based physical activity and fitness program	Andersen Test (10 min 10-m intermittent shuttle run)
Faigenbaum et al. (2015) [43]	United States	*n* = 21 (control) *n* = 20 (intervention)	Fourth grade children	Eight weeks	RCT; single component; integrative strength skill and fitness program during physical education	20-m PACER
Harrell et al. (1996) [44]	United States	*n* = 686 (control) *n* = 588 (intervention)	Rural and urban third and fourth graders	Eight weeks	RCT; multicomponent; exercise program, nutrition and smoking education classes	Eurofit submaximal cycle ergometry test
Harrison et al. (2006) [45]	Ireland	*n* = 130 (control) *n* = 182 (intervention)	Low-income primary school-aged children	16 weeks	Non-RCT; single component; health education curriculum	20-m multistage fitness test
Katz et al. (2010) [46]	United States	*n* = 503 (control) *n* = 603 (intervention)	Elementary school-aged children	Eight months	Non-RCT; single component; academic classroom activity breaks	20-m PACER
Lau et al. (2016) [47]	China	*n* = 40 (control) *n* = 40 (intervention)	Primary school-aged children	12 weeks	RCT; single component; active video game intervention	20-m PACER
Magnusson et al. (2012) [48]	Iceland	*n* = 151 (control) *n* = 170 (intervention)	Elementary school-aged children	24 months	RCT; multicomponent; increasing physical activity, promotion of active commuting, outdoor teaching, healthy dietary promotion	Maximal progressive cycle ergometer test
McKenzie et al. (1996) [49]	United States	*n* = 1294 (control) *n* = 1920 (intervention)	Elementary school-aged children	30 months	RCT; multicomponent; health-related physical education, teacher training, on-site consultation	Nine-minute distance run
Meyer et al. (2014) [50]	Switzerland	*n* = 100 (control) *n* = 181 (intervention)	Elementary school-aged children	Nine months	RCT; multicomponent; additional physical education classes, academic classroom activity breaks, physical activity homework	20-m multistage fitness test
Nogueira et al. (2014) [51]	Australia	*n* = 75 (control) *n* = 76 (intervention)	Primary school-aged girls	Nine months	Non-RCT; single component, addition of 10-min exercise sessions three times per week	20-m multistage fitness test
Reed et al. (2008) [52]	Canada	*n* = 81 (control) *n* = 156 (intervention)	Elementary school-aged children	16 weeks	RCT; multicomponent; school environment, scheduled physical education, extra-curricular, school spirit, family and community, classroom physical activity	20-m multistage fitness test
Resaland et al. (2011) [53]	Norway	*n* = 86 (control) *n* = 102 (intervention)	Primary school-aged children	24 months	Non-RCT; single component; additional 60 min of physical activity during school hours	Maximal progressive treadmill test
Sallis et al. (1997) ^†^ [54]	United States	*n* = 360 (control) *n* = 595 (intervention)	Elementary school-aged children	24 months	RCT; multicomponent; health-related physical education, self-management	One-mile run test
Sollerhed and Ejlertsson (2007) [55]	Sweden	*n* = 74 (control) *n* = 58 (intervention)	Rural primary school aged children	36 months	Non-RCT; single component; expanded physical education lessons	Six-minute running test
Taylor et al. (2018) [56]	United Kingdom	*n* = 55 (control) *n* = 60 (intervention)	Primary school-aged children	Eight weeks	RCT pilot; multicomponent; active breaks, videos, running clubs, playground challenges, teacher training, newsletters and activity homework	20-m multistage fitness test
Thivel et al. (2011) ^†^ [57]	France	*n* = 228 (control) *n* = 229 (intervention)	Primary school-aged children	Six months	RCT; single component; additional two hours of exercise per week	20-m multistage fitness test

Note: RCT stands for randomized control trial; PACER stands for Progressive Aerobic Cardiovascular Endurance Run; ^†^ denotes a study where the reporting of the results was stratified; reported sample sizes are the number of children that completed the respective cardiorespiratory endurance assessment and subsequently used for analysis within each study.

**Table 2:** Fixed effect parameter estimates from random effects meta-regression.

	B-Coefficient	95% Confidence Interval	*p*-Value
Multi-Component	−0.09	−0.40–0.23	0.560
Length (weeks)	0.001	−0.002–0.004	0.427

Note: Referent for component is single component interventions; length predictor variable is on the continuous measurement scale.

## Supplementary Materials

The following are available online at http://www.mdpi.com/1660-4601/15/8/1764/s1, Table S1. Filled studies from the Trim and Fill methodology. Figure S1. Adjusted Funnel Plot following the trim and fill methodology for cardiorespiratory endurance. Note: Filled studies indicted by square data points.
