# Supplementary material for: Moderators of School-Based Physical Activity Interventions on Cardiorespiratory Endurance in Primary School-Aged Children: A Meta-Regression
Source: Int J Environ Res Public Health. 2018 Aug 16;15(8):1764. doi: 10.3390/ijerph15081764 (PMC6121563; doi:10.3390/ijerph15081764)
Supplement: Supplementary file 1 [file ijerph-15-01764-s001.zip › Table S1.pdf]

Table S1. Filled studies from the Trim and Fill methodology.

	Fixed Effects Weight	Random Effects Weight	SMD	95% CI
Fill 1	27.6	8.3	-0.95	-1.32, -0.57
Fill 2	47.3	9.5	-0.82	-1.10, -0.53
Fill 3	18.6	7.3	-0.54	-1.00, -0.09
Fill 4	27.2	8.3	-0.42	-0.80, -0.05
Fill 5	44.8	9.4	-0.34	-0.63, -0.04
Fill 6	86.1	10.4	-0.26	-0.47, -0.05
Fill 7	36.9	9.00	-0.20	-0.52, 0.13

Note: SMD stands for standardized mean difference; 95% CI stands for 95% Confidence Interval.
