# Supplementary figures and images for: Moderators of School-Based Physical Activity Interventions on Cardiorespiratory Endurance in Primary School-Aged Children: A Meta-Regression
Source: Int J Environ Res Public Health. 2018 Aug 16;15(8):1764. doi: 10.3390/ijerph15081764 (PMC6121563; doi:10.3390/ijerph15081764)

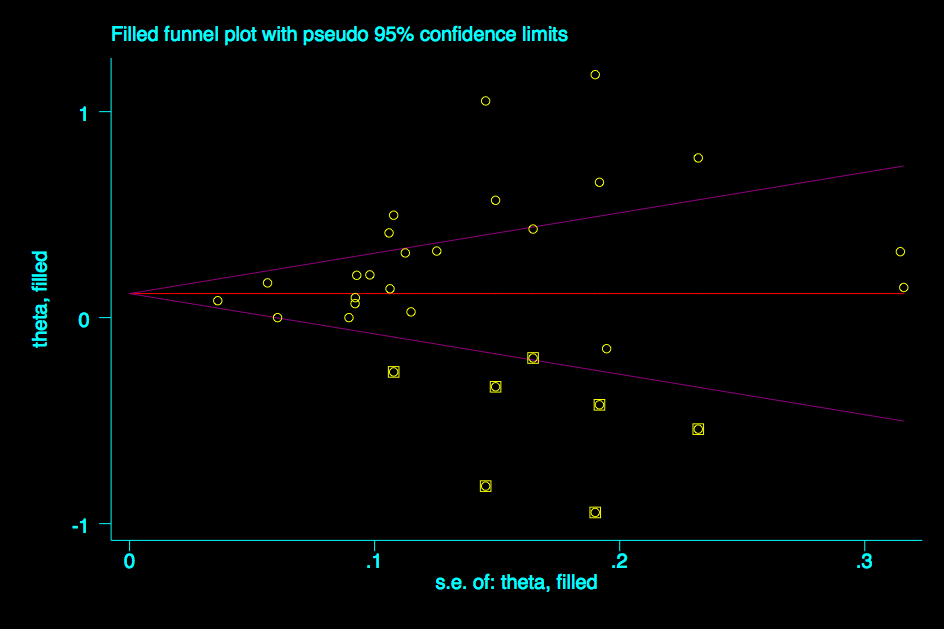

Supplement: Supplementary file 1 [file ijerph-15-01764-s001.zip › Figure S1.jpg]
